# Evaluation of the optical and biomechanical properties of bioengineered human skin generated with fibrin-agarose biomaterials

**DOI:** 10.1117/1.JBO.25.5.055002

**Published:** 2020-05-07

**Authors:** Ana Maria Ionescu, Jesus Chato-Astrain, Juan de la Cruz Cardona, Fernando Campos, Maria M. Pérez, Miguel Alaminos, Ingrid Garzón

**Affiliations:** a University of Granada, Laboratory of Biomaterials Optics, Department of Optics, Faculty of Sciences, Granada, Spain; b Instituto de Investigación Biosanitaria ibs.GRANADA, Granada, Spain; c University of Granada, Department of Histology, Faculty of Medicine, Tissue Engineering Group, Granada, Spain

**Keywords:** optical properties, absorption, scattering, bioengineered skin, fibrin-agarose biomaterial

## Abstract

**Significance:** Recent generation of bioengineered human skin allowed the efficient treatment of patients with severe skin defects. However, the optical and biomechanical properties of these models are not known.

**Aim:** Three models of bioengineered human skin based on fibrin-agarose biomaterials (acellular, dermal skin substitutes, and complete dermoepidermal skin substitutes) were generated and analyzed.

**Approach:** Optical and biomechanical properties of these artificial human skin substitutes were investigated using the inverse adding-doubling method and tensile tests, respectively.

**Results:** The analysis of the optical properties revealed that the model that most resembled the optical behavior of the native human skin in terms of absorption and scattering properties was the dermoepidermal human skin substitutes after 7 to 14 days in culture. The time-course evaluation of the biomechanical parameters showed that the dermoepidermal substitutes displayed significant higher values than acellular and dermal skin substitutes for all parameters analyzed and did not differ from the control skin for traction deformation, stress, and strain at fracture break.

**Conclusions:** We demonstrate the crucial role of the cells from a physical point of view, confirming that a bioengineered dermoepidermal human skin substitute based on fibrin-agarose biomaterials is able to fulfill the minimal requirements for skin transplants for future clinical use at early stages of *in vitro* development.

## 1 Introduction

As the largest organ of the human body and the protective barrier from the surrounding environment, the human skin guards the underlying organs and protects the body against pathogens and microorganisms. However, multiple diseases and conditions, including tumors, ulcers, infections, burns, and trauma among others, can affect human skin. Nowadays, surgical reconstruction or replacement for the damaged skin is the chosen treatment of different skin pathologies. In severe cases, multiple surgical procedures may be needed in a short period of time.^1^^,^^2^ However, the lack of sufficient autologous donor skin areas in patients with large skin burns and other severe conditions makes necessary to find alternative sources of human skin for clinical use. Using advanced tissue engineering approaches, human skin substitutes have been generated in laboratory and used for clinical applications.^3^^–^^5^

In most cases, natural skin substitutes consist of cultured allogeneic or autologous cell suspensions or sheets, which are used on their own or along with a dermal matrix. A great number of the currently available human skin substitutes use type I collagen, collagen-chitosan, fibrin, and other biomaterials.^6^^,^^7^ However, several drawbacks and problems can appear when using most of these artificial skin models, such as contraction of collagen scaffolds^8^ and the low consistency and difficult surgical handling of the autologous fibrin-based scaffolds.^9^ In this context, over the past few years, an innovative model of human skin substitute based on fibrin-agarose biomaterials was developed.^10^ The structure and histological architecture of this model was able to reproduce the native human skin, especially after long-term of *in vivo* implantation.^10^

The optical properties of the human skin are of high relevance, as complex optical interactions of the many different skin components with light provide the final appearance of this organ. The visual appearance of normal skin is determined by several factors, including scattering to incident light, melanin content and blood volume fraction in the different layers of the skin, among others.^11^^,^^12^ The absorption coefficient $\mu_{a}$, the scattering coefficient ${\mu_{s}}^{\prime }$, and chromophore concentrations of skin are fundamental optical properties of the human skin. However, these properties could be different for each layer of the human skin^13^ and determining these parameters in each layer and in the whole organ will provide us with essential information for aesthetic, therapeutic, and diagnostic applications.^14^ Melanin and hemoglobin, two important components of skin, are mainly responsible for the absorption of light by skin in the visible wavelength range. In addition, the absorption by melanin decreases steadily with wavelength, resulting in a color that is rich in red and poor in blue. Absorption by other elements such as cells or fibers seems to be negligible.^15^ In consequence, knowledge of the optical properties of the human bioengineered skin models generated using the principles of tissue engineering represents a fundamental step toward the fabrication of a complete bioengineered human skin able to mimic the native human skin. Bioengineered tissues should be able to resemble the native tissues, not only at the histological level but also for the optical and biomechanical properties. In fact, the biomechanical properties are fundamental to guarantee that the bioengineered skin will be fully functional once grafted *in vivo*, since the clinical performance and the appearance of this skin will be directly affected by the optical and biomechanical properties of the biomaterials involved in its fabrication.^16^^–^^18^ A proper characterization of bioengineered human tissues generated in the laboratory as advanced therapy medicinal products is a crucial requirement of all National Medicines Agencies before clinical use.^19^ Characterization must be fulfilled at several levels to guarantee the appropriateness of the bioartificial tissue, and this may include biomechanical and optical characterization of tissues exposed to the external environment such as the human skin.

This work aims to evaluate the optical and biomechanical properties of a bioengineered human skin model based on fibrin-agarose scaffolds as skin substitutes. These characteristics of this new skin model are essential to optimize its design and possible clinical performance and usefulness.

## 2 Materials and Methods

### 2.1 Tissue Samples and Cellular Isolation

Normal human skin biopsies were collected from healthy donors in order to obtain primary cell cultures of human epidermal (keratinocytes) and dermal cells (fibroblasts). To obtain human keratinocytes, biopsies were carefully rinsed in phosphate-buffered saline and the explant technique was used to obtain small fragments of epidermal tissue. Tissue fragments were seeded on culture flasks and incubated overnight at 37°C with 1 ml of keratinocyte culture medium to favor explant attachment to the culture surface as previously reported.^20^^,^^21^ Subsequently, 1 ml of medium was added every day until a final volume of 5 ml. Keratinocyte culture medium consists of a 3:1 mixture of Dulbecco’s modified Eagle’s medium (DMEM) and Ham’s F12 culture medium supplemented with 10% fetal calf serum, 1% antibiotics, 24  μg/ml adenine, 0.4  mg/ml hydrocortisone, 5  mg/ml insulin, 10  ng/ml epidermal growth factor, and 1.3  ng/ml triiodothyronine (Sigma-Aldrich, USA). Dermal fragments from the skin biopsies were enzymatically digested using 2-mg/ml *Clostridium histolyticum* collagenase I (Gibco-BRL) at 37°C for 6 h to obtain primary cell cultures of skin fibroblasts following previously described protocols.^10^^,^^22^ Isolated fibroblasts were collected by centrifugation and expanded in 25-mm culture flasks containing basal cell culture medium (DMEM supplemented with 10% fetal bovine serum and 1% antibiotics) under standard cell culture conditions.

### 2.2 Generation of Fibrin-Agarose Hydrogels

Once primary cell cultures of human skin keratinocytes and fibroblasts were obtained, bioengineered tissues were generated using fibrin-agarose biomaterials following previously published methods.^10^^,^^23^^–^^27^ Briefly, human plasma was used as a source of fibrin and 0.1% agarose, tranexamic acid, and calcium chloride were added and immediately aliquoted on culture plates. Four types of samples were used in this study: (1) acellular fibrin-agarose substitutes (AS), in which fibrin-agarose biomaterials were generated without cells; (2) dermal skin (DS) substitutes consisting of 800,000 human skin fibroblasts immersed within fibrin-agarose biomaterials; (3) full-thickness dermoepidermal skin substitutes (EDS) consisting of 800,000 human skin fibroblasts immersed within fibrin-agarose biomaterials (as in the DS group), with 500,000 human skin keratinocytes subcultured on top of the biomaterial to develop a epidermal layer. EDS samples contained both skin layers—dermal and epidermal layers—unlike DS samples that exclusively consisted of a dermal layer; and (4) human native skin tissue (CTR) used as a control.

For the time-course study, all bioengineered skin substitutes (AS, DS, and EDS) were kept in culture for 7, 14, 21, and 28 days. To promote epidermal differentiation,^28^ the air–liquid culture technique was used during the two last weeks in 21- and 28-days bioartificial tissues. Samples corresponding to each follow-up period (7, 14, 21, and 28 days) were carefully removed from the well plates and subjected to plastic compression techniques according to previously described and standardized procedures.^15^ Briefly, all samples were placed between a couple of nylon filter membranes with 0.22-μm pore size (Merck-Millipore, Darmstadt, Germany) and compressed between a pair of pieces of sterile Whatman 3-mm absorbent paper below a flat glass device. Uniform mechanical pressure (500 g, homogeneously distributed) was applied for 3 min to obtain a high-density membrane-like nanostructured fibrin-agarose hydrogel film with ∼50 to 60  μm thickness in all substitutes (AS, DS, and EDS).

### 2.3 Histological Analysis

All study samples were fixed in 10% buffered formalin, embedded in paraffin, and 5-μm-thick sections were obtained. Sections were stained with hematoxylin and eosin and histologically analyzed using a Nikon Eclipse 90i light microscope.

### 2.4 Optical Properties

**Fig. 1:**
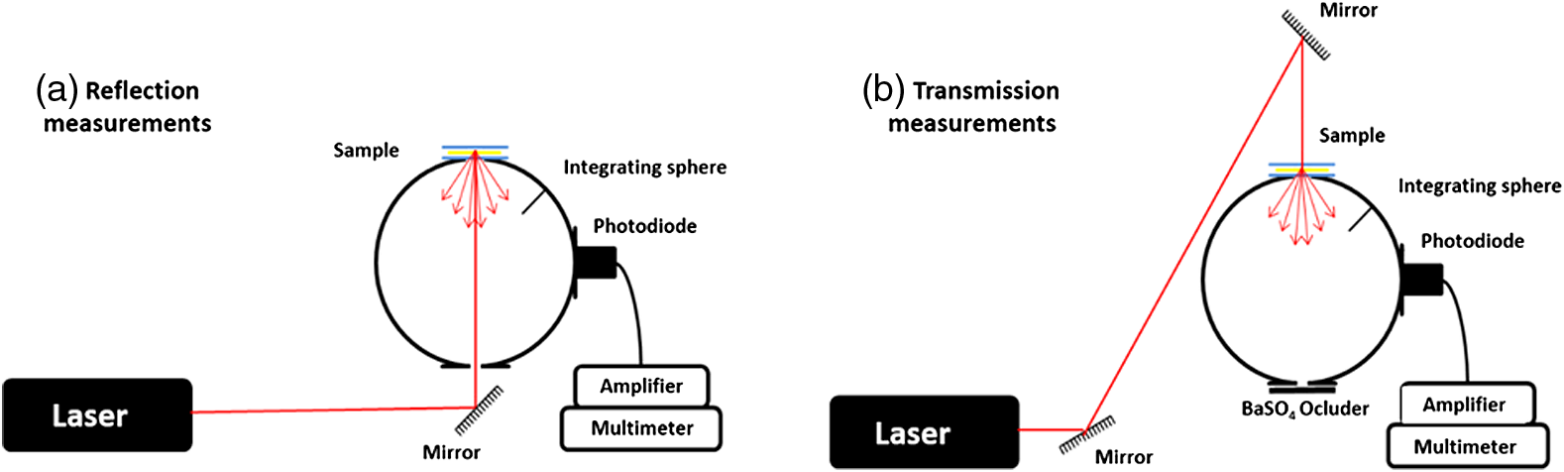
Experimental setup used to measure the optical properties of each sample included in the study. (a) Reflection measurements: to calculate the sample reflectance, the sample was first analyzed, followed by the reflectance standard and finally, a reflection measurement with the empty sample port. (b) Transmission measurements: the sample was first measured, then a measurement with the empty sample port was performed and finally, a measurement with no illumination from the lasers was performed.

Total reflection and total transmission measurements of the bioengineered human skin models and control human skin samples were obtained using a single integrating sphere as previously described by the inverse adding-doubling (IAD) method.^29^^,^^30^ A schematic representation of the experimental setup is shown in Fig. 1.

Briefly, a 158.2-mm-diameter integrating sphere (Oriel, model 70674, USA) with an 11-mm-diameter detector port and a 4-mm-diameter sample port with a baffle between ports and also a 15-mm-diameter entrance port was used for both total reflection and total transmission measurements. An argon ion laser (Stellar-Pro-L Model, Modu-Laser, USA) that provided 457.9, 488, and 514.5 nm wavelengths and a 632.8-nm wavelength He–Ne laser (30564 Model, Research Electro-Optics, USA) were used for all measurements. This discrete selection of wavelengths is in accordance with the methodology used in the literature for the determination of optical properties of human tissues.^29^^,^^31^^–^^37^ Furthermore, the distinctive absorption peaks within the visible region displayed by hemoglobin (one around 450 nm, and the other two between 500 and 600 nm) and the absorption decrease from the ultraviolet to the infrared region displayed by the melanin^38^ (hemoglobin and melanin being the major absorbing molecules in the native skin) make the selection of wavelengths suitable for the evaluation proposed in this study. Other structures showing high scattering at the visible light wavelengths are (1) whole nuclei, (2) organelles such as lysosomes and mitochondria, and (3) small particles such as ribosomes or large protein complexes.^39^

The maximum output power of the lasers was 1000  mW±5% for the argon laser and 12 mW for the He–Ne laser. Both argon and He–Ne lasers beams had 2-mm diameters. Each sample was measured three times using the reflection setup [$R(r_{s}^{\text{direct}},r_{s})$]. These measurements were referenced to a 98% Optopolymer reflectance standard (OPST3-C, Optopolymer, Germany) [$R(r_{\mathrm{std}},r_{\mathrm{std}})]$ and a dark measurement (with the sample port empty) [R(0,0)]. The total reflectance of each sample was then calculated as

$$M_{R}=r_{\mathrm{std}}\frac{R(r_{s}^{\text{direct}},r_{s})-R(0,0)}{R(r_{\mathrm{std}},r_{\mathrm{std}})-R(0,0)},$$

where $r_{\mathrm{std}}$ is the reflectance of the reflectance standard as given by the manufacturer.

For transmission, three measurements were performed on each sample [$T(r_{s}^{\text{direct}},r_{s})$], and they were referenced to 100% with the lasers illuminating the open port (empty port) [T(0,0)] and a dark measurement with an open port but with no illumination from the lasers ($T_{\text{dark}}$). Then, the total transmittance of each sample was calculated as

$$M_{T}=\frac{T(r_{s}^{\text{direct}},r_{s})-T_{\text{dark}}}{T(0,0)-T_{\text{dark}}}.$$

For each sample, measurements were repeated three times and the obtained values agreed within 5%.

The reflectance of the sphere wall and the sample thickness were also measured and taken into account when calculating the absorption and reduced scattering coefficients. These calculations were performed using the available MC software for IAD method developed by Prahl et al.^30^ This method has been widely used for processing experimental data obtained using integrating spheres and rapidly determines iterative solutions.^31^^,^^32^^,^^36^^,^^40^^,^^41^

In order to determine the absorption and the reduced scattering coefficients, the refractive indices of all samples were calculated using the Cauchy dispersion equation, as proposed by Ding et al.^41^ for human skin. Based on the results obtained by previous authors^33^^,^^42^^,^^43^ who found that the range of the anisotropy coefficient for biological skin tissues is 0.71 to 0.95, the average scattering anisotropy coefficient of both artificial and control human skin was assumed to be 0.9, as only measurements of the diffuse reflection and transmission were performed on the samples studied.

### 2.5 Biomechanical Properties

All experimental groups (AS, DS, EDS, and CTR) were subjected to tensile tests using an electromechanical material testing instrument (Instron, Model 3345-K3327).^44^ For this test, the skin substitutes were sectioned to a regular rectangular shape. All experimental groups were oriented with their length along the direction of tension and clamped at each end, leaving a constant distance of 1 cm between the clamps (in all cases 1 cm of the sample was gripped between each clamp). The tests were run at a constant strain rate of 5  mm/min at room temperature. Young’s modulus was calculated as the tangent modulus of the initial, linear portion of the stress–strain curve of each experimental run, while the stress at break (σ break) and the strain at break (ε break) values were determined by selecting the point of the stress–strain curve where the fracture occurred. A 50-N Instron load cell was used to obtain the data for the stress–strain curves. Calculation of the average value and standard deviation (SD) of the results for each experimental run was operated automatically, using Instron Blue Hill 2 Material Testing software.

### 2.6 Statistical Analysis

In the first place, we obtained the averages and SDs for each global group of samples: (1) each type of sample—AS, DS, EDS, and CTR—independently of the culture time (all samples corresponding to the same type were considered together regardless the culture time), and (2) each development time—7, 14, 21, and 28 days—independently of the sample type (all samples corresponding to the same time were considered together). Then, averages and SDs were acquired for each specific group of samples (for example, EDS kept in culture for 21 days). All these values were obtained for each variable of the study (reflectance, transmittance, absorption, and reduced scattering coefficients for the optical analysis and Young’s modulus, break load, traction deformation, stress at fracture-break, and strain at fracture break for the biomechanical analysis).

In the second place, the Shapiro–Wilk test was used to determine if each distribution was normally distributed. As none of the variables considered in this work fulfilled the criteria for parametric statistics, nonparametric tests were used for all statistical comparisons. To identify statistical differences between global or specific groups, we used the Mann–Whitney test. This statistical test was used to compare (1) each specific or global group of samples versus control human skin, (2) each global group of samples versus other global groups (for example, the global group of AS samples versus the global group of DS samples), and (3) each specific group of samples versus other specific groups (for example, EDS samples corresponding to 7 days of culture versus EDS samples corresponding to 14 days of culture). Statistical p values were represented in a heat map showing different colors for different values of statistical significance. To determine whether the behavior of one variable is correlated or not with that of another variable, we used Kendall’s *tau* correlation test. Comparisons were carried out with SPSS 16.00 software, and p values below 0.05 were considered statistically significant in two-tailed tests.

## 3 Results and Discussion

### 3.1 Histological Analysis

**Fig. 2:**
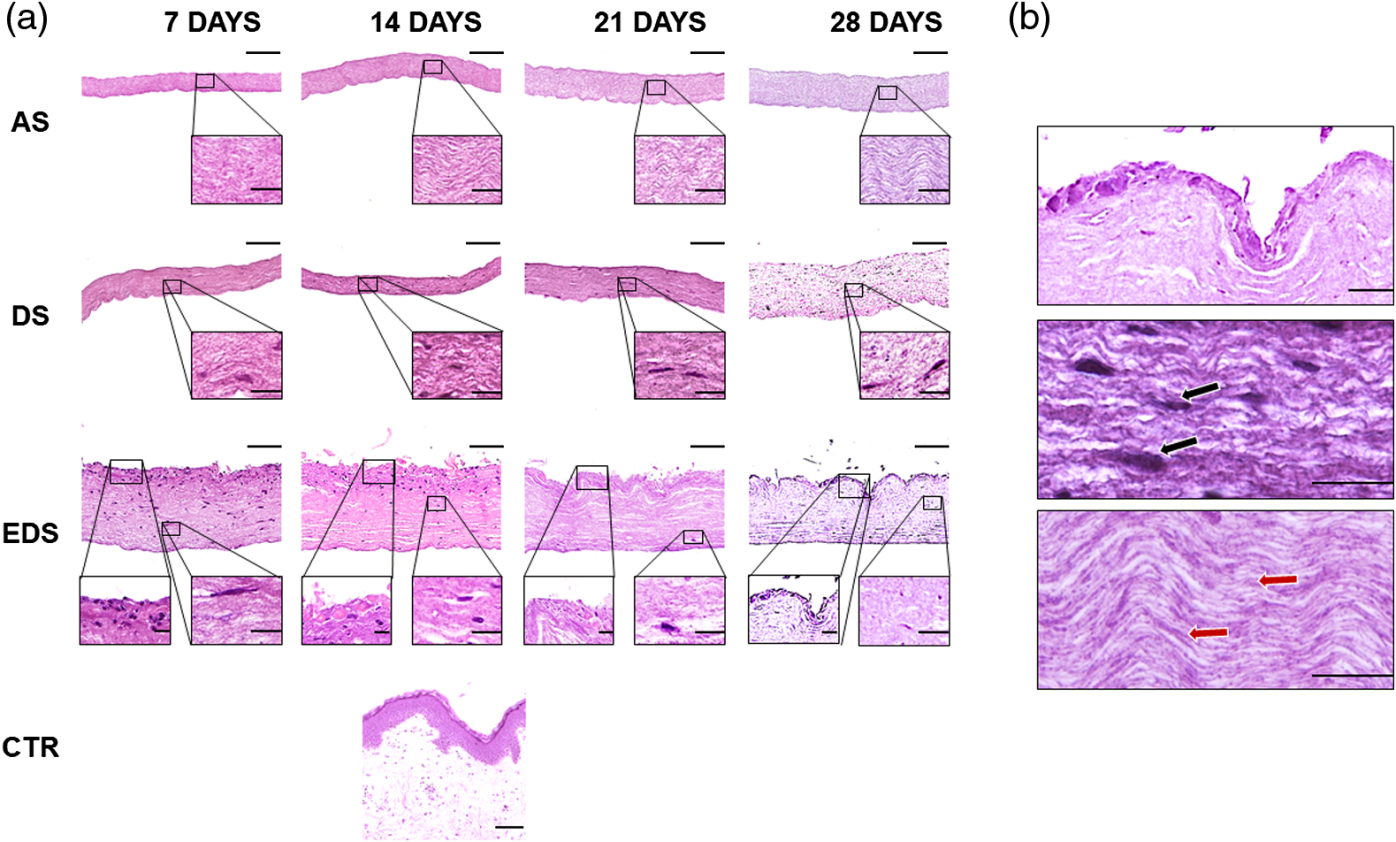
Histological analysis of all samples included in this study. (a) Low and middle magnification images for each specific sample. (b) High magnification images of EDS samples kept in culture for 28 days. AS, acellular fibrin-agarose substitutes; DS, dermal skin substitutes; EDS, full-thickness dermoepidermal skin substitutes; CTR, control native human skin. Black arrows point to some cells immersed in the biomaterial; red arrows correspond to parallel fibers of the biomaterial after the nanostructuration process. Scale bar: 100  μm in low-magnification images in (a) and 20  μm in middle magnification insets in (a) and figures in (b).

The histological analysis of all three types of fibrin-agarose samples (shown in Fig. 2) revealed a high-density membrane-like structure after the nanostructuring process. In all cases, the biomaterial fibers in the dermal substitute tended to appear oriented, and fibers acquired a parallel orientation. These findings confirm the idea that plastic compression was able to expel excess fluid from the originally hyperhydrated fibrin-agarose hydrogel, thus modifying the biomaterial density and alignment.^45^ For dermal and dermoepidermal substitutes (DS and EDS), a mild cell density was observed inside the dermal substitute in all study times. In the EDS group, we found up to three layers of epidermal keratinocytes. The analysis of the bioengineered human skin substitutes showed that the fibroblasts had a proper development in the fibrin-agarose biomaterial and promoted the epidermal differentiation in EDS, demonstrating that the underlaying dermal layer, along with the epidermal layer, plays an orchestrated role in cell proliferation and differentiation of both the dermal and the epidermal layers of the skin.^46^

### 3.2 Optical Properties

The appearance of human skin fundamentally depends on the absorption, as well as the scattering of the light that propagates through it.^17^ Therefore, the absorbing and scattering properties of bioengineered human skin models should be similar to the native tissue in order to create a homogeneous appearance once implanted *in vivo*. Since no direct measurement methods are available to determine the absorption and scattering coefficients of biological media, an iterative method has been used in this work. This method was based on the IAD method involving direct measurements of reflection and transmission of the samples (using a single integrating sphere), and a Monte Carlo simulation used to calculate the absorption and scattering coefficients.^29^^,^^30^

**Fig. 3:**
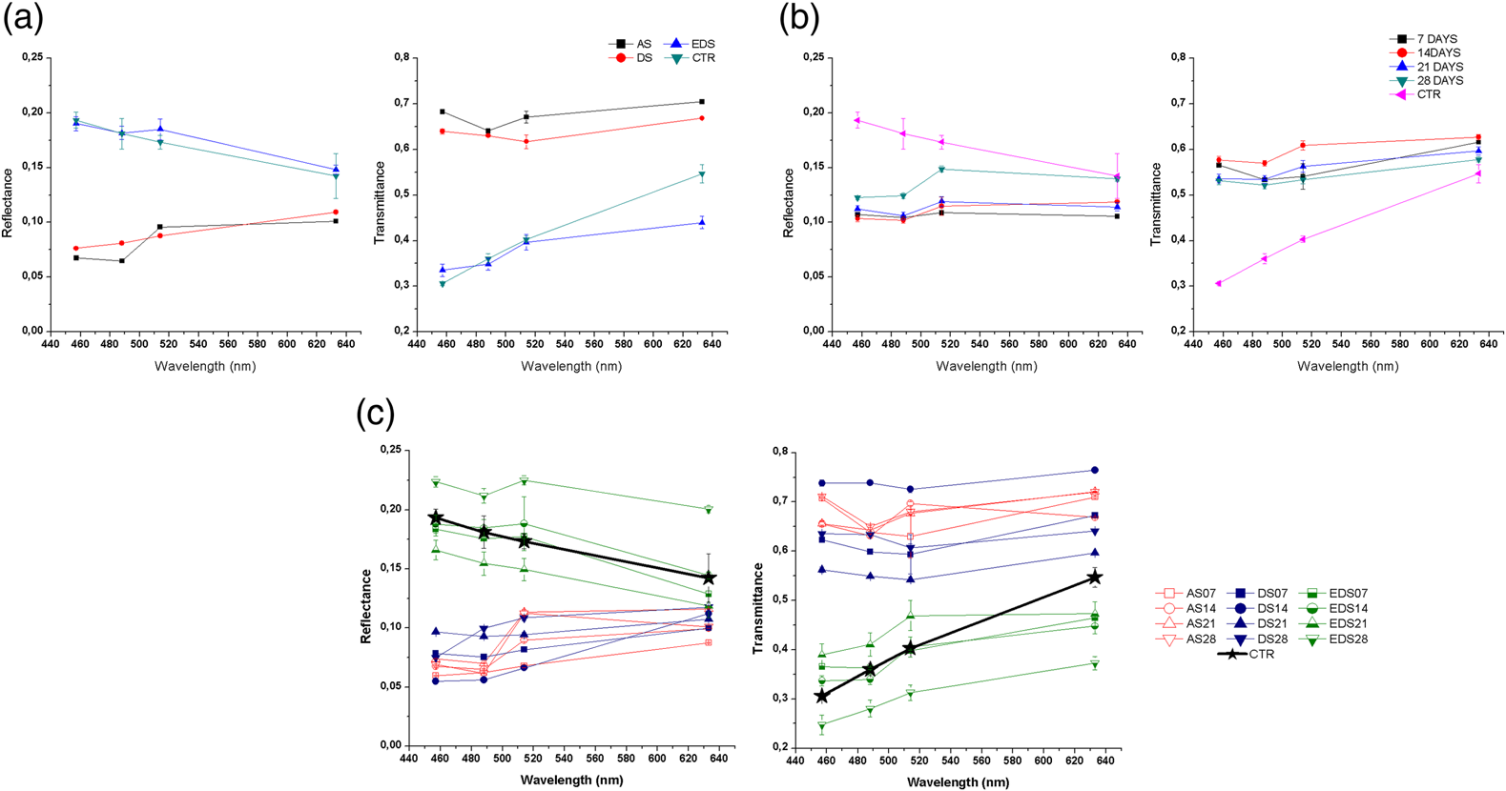
(Left) Reflectance and (right) transmittance values of (a) the global groups of samples, (b) global groups of times in culture, and (c) specific samples.

The total diffuse reflectance and diffuse transmittance values of each type of bioengineered skin (AS, DS, and EDS) were analyzed weekly up to 28 days in culture and compared to native human skin. Results of these analyses are shown in Fig. 3. First, we found that the reflectance spectral behavior of the AS global samples (all AS tissues regardless the culture time) was similar to DS global samples, with increasing reflectance values as the wavelength increased [Fig. 3(a), left]. In contrast, the EDS global group displayed a different spectral behavior that was similar to control native skin, showing that the reflectance values decreased with increasing wavelength (differences between EDS and CTR were nonsignificant at 488 nm). Regarding time in culture [Fig. 3(b), left], the spectral behavior of CTR samples showed higher reflectance than the rest of the global samples (p<0.001), and bioengineered tissues corresponding to 28 days were higher than global samples at 7, 14, and 21 days of development (p<0.001). When specific samples were analyzed [Fig. 3(c), left], we found that the highest reflectance values were obtained for the EDS models after 28 days of development in culture (p<0.001). However, these values were higher than those of the control samples. The bioengineered models that most resembled the reflectance spectral behavior and values of the native tissue were the EDS after 7 to 14 days of development in culture, with no statistically significant differences with CTR (p>0.05).

Regarding the capability of the artificial skin to transmit the incoming light, the AS and DS global groups of samples showed a similar pattern, which significantly differed (p<0.001) from EDS and CTR, which were very similar and reached lower values than AS and DS [Fig. 3(a), right]. For the global time groups, all times showed a similar behavior as shown in Fig. 3(b), right. Analysis of the specific groups confirmed that AS and DS models showed the highest diffuse transmittance values, which were higher than CTR and EDS specific groups [Fig. 3(c), right], suggesting that the presence of an epithelium on top of the human bioengineered DS influenced the transmittance spectral behavior, thus causing a decrease in the transmittance values for all wavelengths and all the studied culture times. Strikingly, DS samples corresponding to short development times tended to show higher transmittance than long time periods. Most likely, this phenomenon could be explained by the fact that bioengineered connective tissues, including the dermal substitutes generated in this work, tend to synthetize collagen, proteoglycans, and other extracellular matrix component in a time-dependent manner, and these components may influence the optical properties of these artificial tissues.^47^ For the EDS models, the transmittance values increased at 21 days, and a decrease was observed at day 28. Nevertheless, the EDS group displayed a similar transmittance behavior to the control samples, with values increasing as the wavelength increased. Once again, the bioengineered models that most resembled the transmittance spectral behavior and values of the native tissue were the EDS after 7 to 14 days of development in culture, with no statistically significant differences with the transmittance of the native control skin (p>0.05).

**Fig. 4:**
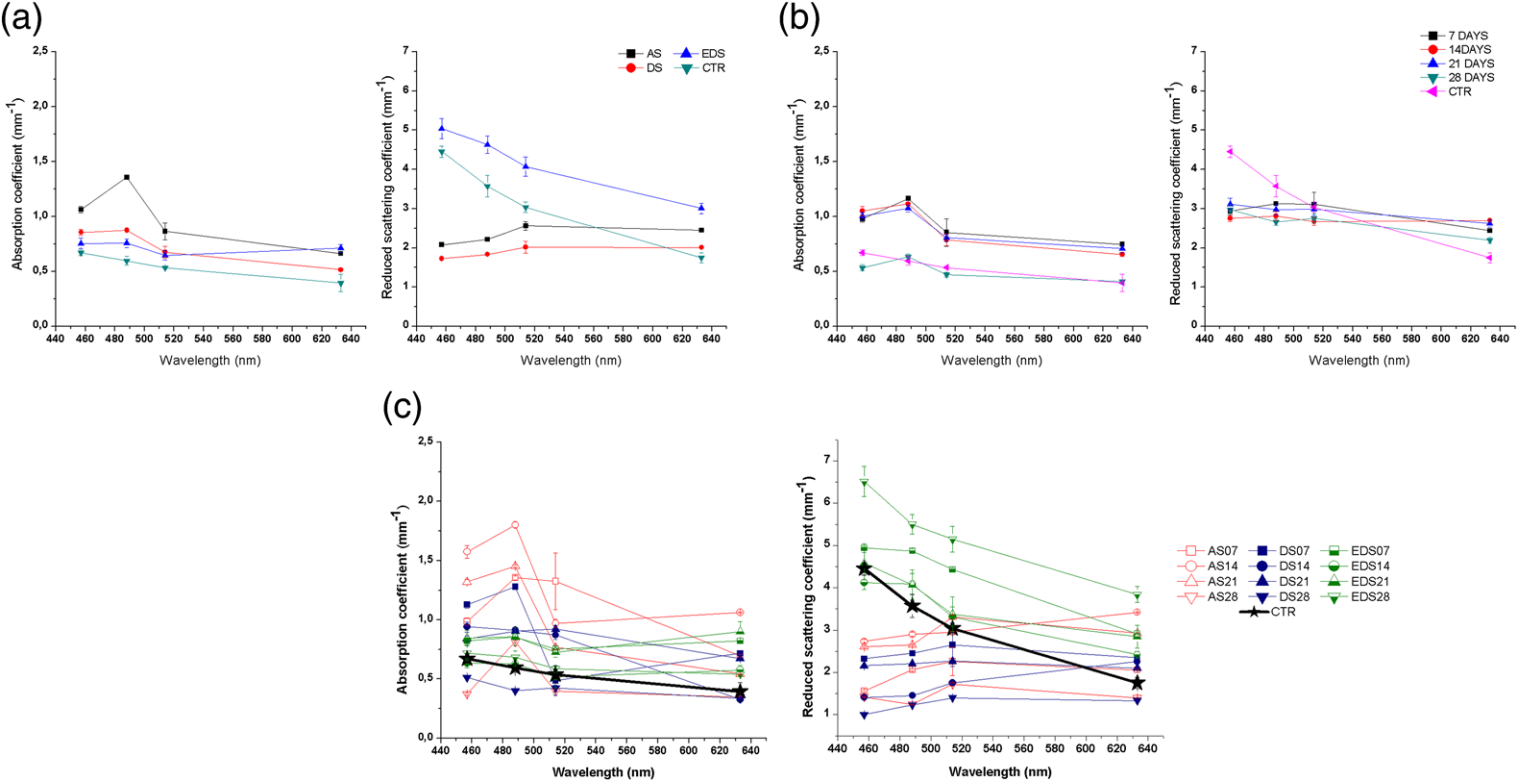
(Left) Absorption and (right) reduced scattering coefficients of the (a) global groups of samples, (b) global groups of times in culture, and (c) specific samples.

In order to explain these reflectance and transmittance patterns, we then analyzed the absorption and reduced scattering values of the AS, DS, and EDS groups and controls using the IAD method, since these are among the main factors that may influence these parameters. Considering an anisotropy factor g=0.9, according to the calculations made with the available IAD software, the errors were <6% in the case of the reduced scattering coefficient and <8% in the absorption coefficient case for the AS and DS substitutes. Whereas EDS substitutes, these errors were <2% in the case of scattering and <5% in the case of absorption, with similar trends as the control sample. In consequence, for the absorption, we found very low values in all samples, with all global groups of samples showing significantly higher absorption than CTR (p<0.001) [Fig. 4(a), left]. For the study of the influence of time [Fig. 4(b), left], the global time group corresponding to 28 days showed significantly lower values than the other time groups (p<0.001). Nevertheless, the model that most resembled the absorption coefficient behavior and values of the control tissue was the EDS model, especially in the short wavelength spectrum range analyzed here [Fig. 4(c), left]. Regarding the reduced scattering coefficient, the spectral behavior of AS and DS was very similar, whereas EDS global groups were more comparable to CTR [Fig. 4(a), right]. As shown in Fig. 4(b), right, the influence of the time in culture did not have a clear influence on the scattering values. For the specific groups, we found that the spectral behavior of all EDS groups was different to AS and DS specific groups, and their values were higher than CTR, AS, and DS, especially for the lowest wavelengths [Fig. 4(c), right]. However, significant differences were found between EDS groups and controls. Nevertheless, the reduced scattering coefficient values of the complete fibrin-agarose skin model (EDS) after 14 days of development in culture were in the range of the human native skin found by other authors (Table 1).^38^^,^^48^^–^^51^ In addition, the decrease in the scattering properties as compared to AS and DS groups could explain the higher transmittance values found in EDS, especially for the largest wavelengths. For all sample groups, weeks in culture and wavelengths studied, statistically significant differences with control skin were found (p<0.05) for the reduced scattering.

**Table 1:** Main optical properties determined in this study and published in the available literature.

	λ (nm)	$\mu_{a}$ (${\mathrm{mm}}^{-1}$)	$\mu_{s}^{\prime }$ (${\mathrm{mm}}^{-1}$)	
EDS (7 days)	457	0.82±0.03	4.94±0.10	This study
	488	0.85±0.01	4.87±0.05	
	514	0.75±0.01	4.43±0.05	
	633	0.82±0.01	2.90±0.01	
EDS (14 days)	457	0.63±0.04	4.12±0.17	
	488	0.63±0.01	4.08±0.25	
	514	0.52±0.09	3.31±0.23	
	633	0.58±0.04	2.42±0.08	
EDS (21 days)	457	0.85±0.06	4.58±0.40	
	488	0.86±0.08	4.06±0.36	
	514	0.73±0.04	3.38±0.41	
	633	0.90±0.08	2.85±0.27	
EDS (28 days)	457	0.72±0.06	6.51±0.36	
	488	0.68±0.05	5.50±0.23	
	514	0.56±0.03	5.15±0.30	
	633	0.54±0.02	3.84±0.19	
Control skin sample	457	0.67±0.02	4.45±0.15	
	488	0.60±0.04	3.57±0.28	
	514	0.54±0.01	3.04±0.13	
	633	0.39±0.08	1.75±0.13	
Stratum corneum	450	1.16	4.52	Ref. 47
	500	1.05	4.19	
	550	0.98	3.87	
	650	0.82	3.22	
Piglet skin (epidermis + dermis)	630	0.1±0.01	2.27±0.08	Ref. 48
Caucasian skin	400	1.35	3.43	Ref. 49
	500	0.62	2.51	
	600	0.38	1.87	
Epidermis	450	1	9	Ref. 50
	500	0.7	7	
	550	0.45	6	
	630	0.25	5	
Dermis	450	0.6	6	
	500	0.35	4.5	
	550	0.25	3.5	
	630	0.15	3	

Considering all the results obtained for the optical properties, it can be stated that scattering is the most important optical phenomenon that affects the propagation of radiation through the tissue, especially in the EDS models of full-thickness bioengineered skin with epithelium on top. This was previously demonstrated for other native tissues such as the human oral mucosa and cornea,^36^^,^^47^^,^^52^ suggesting that the epithelial layer of these tissues may play a crucial role not only as a mechanical protective barrier but also as a filter for the incoming light. The reason why the epithelial layer may show these scattering levels could probably be related to the high concentration of intracellular components, such as nuclei, organelles, and small particles, that are present in this type of tissues and the high scattering properties of these components.^39^ Especially, the primary sources of particulate scatter in the visible region within the skin are filamentous proteins.^38^ Keratins are intermediate filamentous proteins of the epidermis and are among its most important constituents, whereas collagen and other ECM proteins are found at the dermal layer.^53^ Further scatter is attributed to melanosomes in the epidermis, cell nuclei, cell walls, and many other structures in the skin that occur in smaller numbers.^54^ In this study, it has been confirmed that the bioengineered skin substitutes containing cells and organized collagen fibrils showed higher absorbing and scattering properties than the acellular constructs, resulting in a lower transmission of light. This could be positive for the barrier function of the human skin, although future *in vivo* studies should confirm this statement using visual and ultraviolet light spectrum wavelengths. The optical behavior of each type of bioengineered skin should be considered as a very important factor before clinical use, since the human skin should have a normal appearance and behavior when visible and ultraviolet light reach the skin, especially in skin areas that are highly exposed such as the facial skin. Most likely, *in vivo* grafting of the bioengineered human skin will increase the presence of other pigments such as hemoglobin and melanin, which will probably increase absorption in these bioartificial tissues.

### 3.3 Biomechanical Properties

**Fig. 5:**
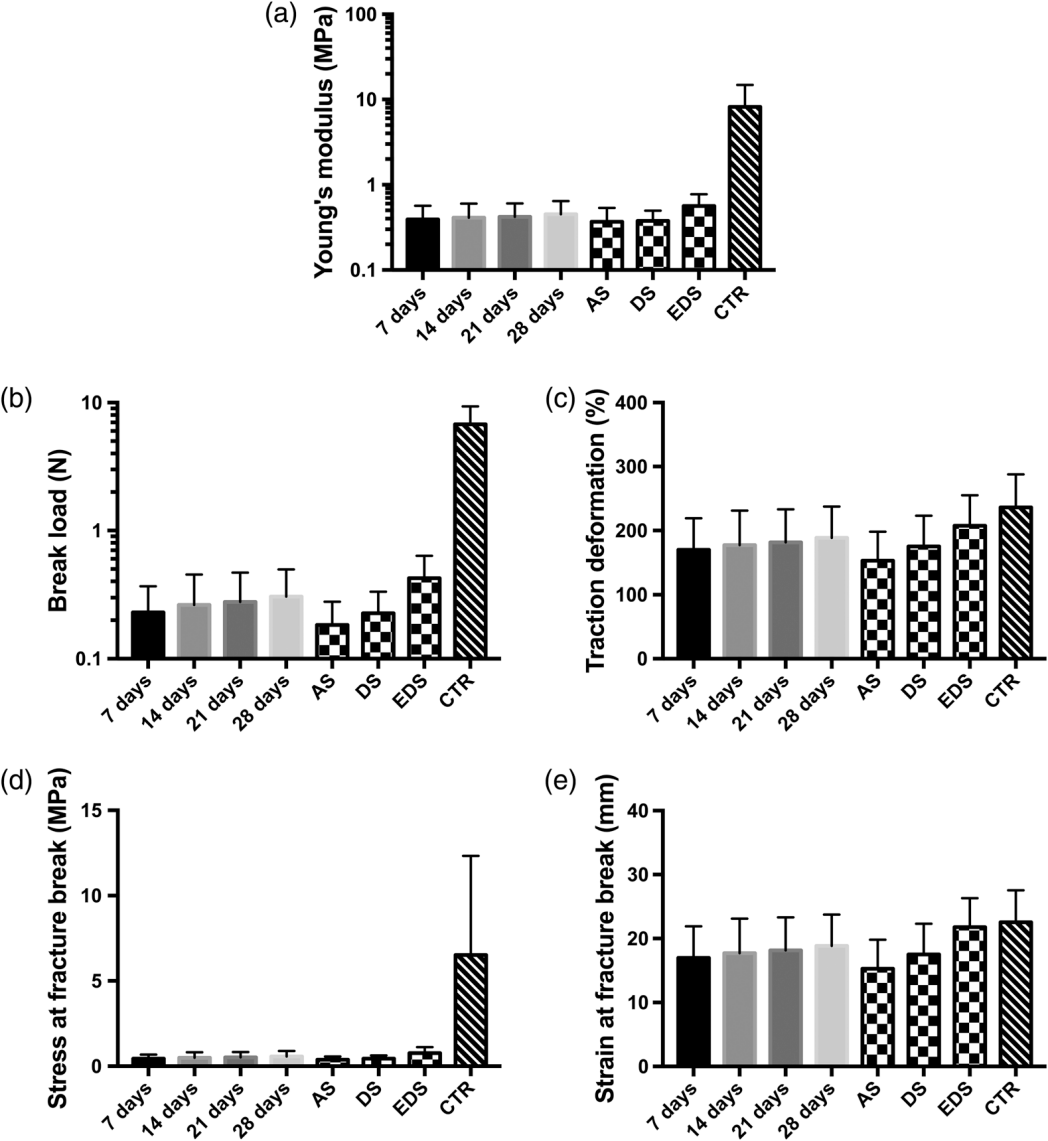
Biomechanical properties corresponding to the global groups of samples (AS, DS, and EDS regardless the culture time, and samples corresponding to days 7, 14, 21, and 28 of culture regardless the sample type) and control human native skin (CTR). (a) Young’s modulus, (b) break load, (c) traction deformation, (d) stress at fracture-break, and (e) strain at fracture break.

To determine the influence of the type of sample and the culture time on the biomechanical properties of the human bioartificial skin, we first analyzed each global group of samples. In this regard, we found that AS samples were very similar to DS for all biomechanical parameters analyzed in this work (p>0.05), whereas EDS showed statistically higher values of all parameters than AS and DS. Strikingly, EDS were similar to CTR for the traction deformation and strain at fracture break, showing statistically lower values of the Young’s modulus, break load, and stress at fracture break (Figs. 5 and 7). A significant correlation was found between the sample type and the five biomechanical parameters analyzed here (p<0.01 for all parameters), suggesting that the type of sample significantly influences the biomechanical behavior of the skin substitutes. Previous reports^55^^,^^56^ demonstrated that the mechanical behavior of artificial substitutes is largely determined by cells, especially by their cytoskeleton network, which contains abundant actin filaments, microtubules, and intermediate filaments,^56^^,^^57^ and the ECM molecules that these cells are able to release to the extracellular medium, and this could explain why full-thickness EDS show better biomechanical properties than DS, and both were higher than acellular AS. These results confirm that cell-enriched substitutes may exert the most biomimetic biomechanical properties and could therefore be used for the treatment of patients with large skin injuries.

Once the relationship between bioengineered samples and biomechanical properties was studied, we carried out a time-course analysis to determine the influence of the culture time on the biomechanical properties. As shown in Figs. 5 and 7, global times groups (7, 14, 21, and 28 days samples regardless the sample type) showed significantly lower values for the Young’s modulus, break load, and stress at fracture break than CTR skin (p<0.01 for all these comparisons), and 21 and 28 days were also lower for the traction deformation and strain at fracture break, whereas 7-day samples were lower than CTR for the traction deformation. No differences were found among the different times for the Young’s modulus, and only some specific differences were detected for some of the parameters. None of the five biomechanical parameters analyzed here showed significant correlation with the time in culture when all samples were considered together (p>0.05). However, specific analysis for each type of sample showed a significant correlation between the traction deformation and the strain at fracture break with the time, only for the EDS group (p=0.044 in both cases). Previous studies^58^ suggest that bioengineered human tissues are able to remodel the biomaterial and synthesize relevant ECM components from days 21 to 28 in culture. The results of this study imply that the biomechanical properties of artificial skin substitutes may be suitable at days 7 to 14 of *in vitro* development.

**Fig. 6:**
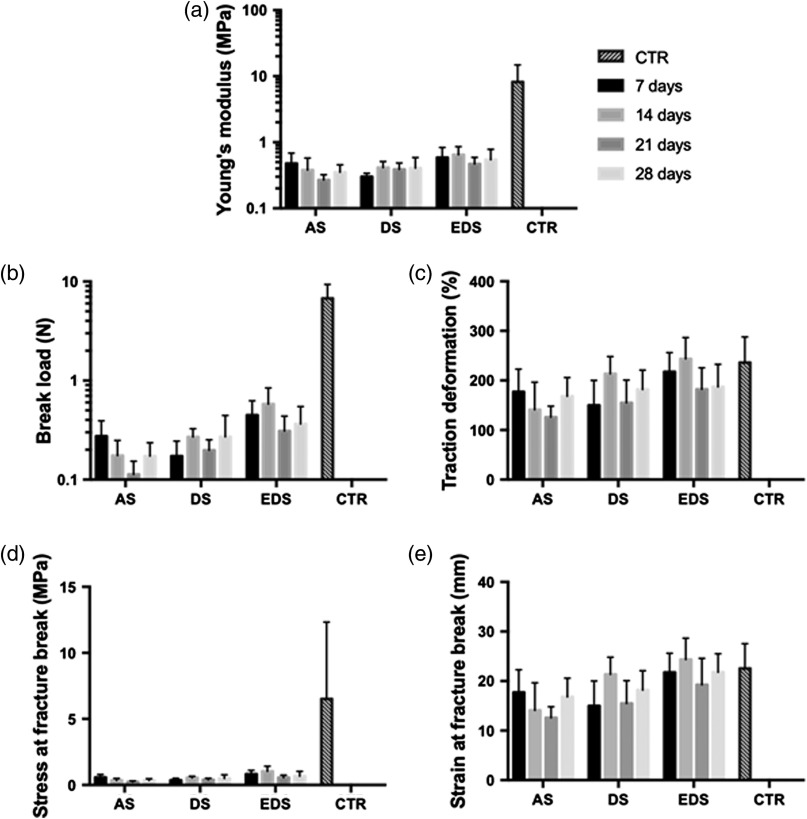
Biomechanical properties of each specific sample (AS, DS, and EDS at days 7, 14, 21, and 28 of development and control native human skin). (a) Young’s modulus, (b) break load, (c) traction deformation, (d) stress at fracture break, and (e) strain at fracture break.

**Fig. 7:**
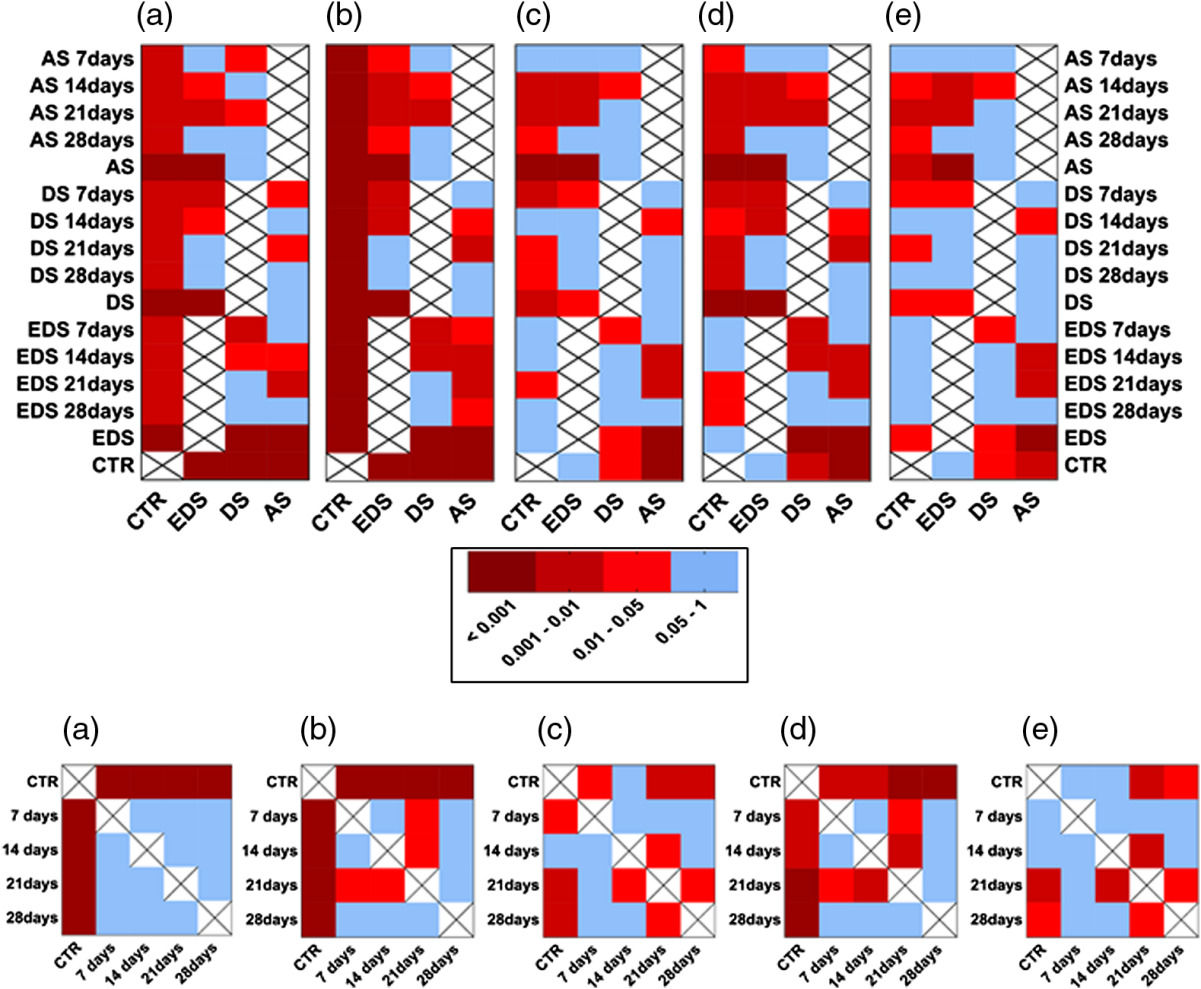
Heat map representing the results of the statistical comparisons made for the biomechanical parameters using the Mann–Whitney nonparametric test. (a) Young’s modulus, (b) break load, (c) traction deformation, (d) stress at fracture-break, and (e) strain at fracture break. Comparisons resulting in statistically nonsignificant p values are shown in blue, whereas statistically significant p values are shown in red, with dark red corresponding to the most significant p values (p<0.001) and light red, to p<0.05.

Once the global groups were analyzed, we wanted to determine the particular behavior of each specific group of samples. As shown in Figs. 6 and 7, the comparison of the biomechanical properties of each specific group of samples versus control native skin showed that some specific times were not statistically different to control samples. Specifically, AS samples at 7 days were not statistically different to controls for traction deformation and strain at fracture (p>0.05), but differences were statistically significant for the rest of culture times. For DS, traction deformation and strain at fracture at 14 days and strain at fracture at 28 days were not statistically different to controls (Fig. 7). For full-thickness EDS samples, the strain at fracture break values was similar to control human skin at all times, whereas the traction deformation was similar to CTR at days 7, 14, and 28. Although the values reached by CTR were very high as compared to the experimental samples, we found that the stress at fracture break of EDS was not statistically different to CTR at days 7 and 14. Most likely, these differences are related to the very low values found in all samples, and to the high SD of CTR samples. Although *in vivo* assays are necessary to determine the biomechanical properties of these substitutes once grafted *in vivo*, these results suggest that the dermoepidermal model of human bioengineered EDS may fulfill the requirements for clinical translation from a biomechanical stand point, reaffirming the idea that the EDS after 7 to 14 days could more adequately resemble the physical properties of the native human skin.

## 4 Conclusions

For the first time, the optical and biomechanical properties of a complete fibrin-agarose human skin substitute have been determined, contributing to a deep characterization of this bioartificial tissue for future clinical use. The absorption and scattering properties, especially this latter one, profoundly modify skin color and will largely determine the light penetration depth into the dermis. The optical properties of the bioengineered skin substitutes evaluated in this study after 7 to 14 days of development in culture, resembled those of native human skin, especially for the dermoepidermal substitutes, suggesting a possible match in the visual appearance of these substitutes and that of the native skin. In addition, the results of this study confirm the importance of the presence of cells in the substitute, especially when dermal and epidermal cells form part of the artificial fibrin-agarose skin substitute and both the dermis and the epidermal layers are differentiated. Although the key role of cells on the biological and physiological functions of the artificial tissue has already been demonstrated, this study clarifies the crucial role of cells from a physical point of view. In addition, results from this study establish that the bioengineered skin fulfills the minimal requirements (in terms of similarity with the visual appearance of native human skin) of artificial tissues for future clinical use at early stages of *in vitro* development (7 to 14 days). This strongly benefits patients with severe and large skin alterations that could be treated in an early fashion due to their critical condition. Future studies should determine if these tissues, corresponding to 1 to 2 weeks of *in vitro* development, are also adequate from a clinical standpoint.
